# Regenerative Endodontic Procedures Using Contemporary Endodontic Materials

**DOI:** 10.3390/ma12060908

**Published:** 2019-03-19

**Authors:** Simone Staffoli, Gianluca Plotino, Barbara G. Nunez Torrijos, Nicola M. Grande, Maurizio Bossù, Gianluca Gambarini, Antonella Polimeni

**Affiliations:** 1 Department of Oral and Maxillo-Facial Science, “Sapienza”-University of Rome, 00100 Rome, Italy; simonestaffoli@hotmail.it (S.S.); bnuneztorrijos@gmail.com (B.G.N.T.); maurizio.bossu@uniroma1.it (M.B.); ggambarini@gmail.com (G.G.); antonella.polimeni@uniroma1.it (A.P.); 2 Department of Endodontics, Catholic University of Sacred Heart, 00100 Rome, Italy; nmgrande@gmail.com

**Keywords:** Immature permanent tooth, necrotic pulp, regenerative endodontics, revascularization, revitalization

## Abstract

Calcium hydroxide apexification and Mineral Trioxide Aggregate (MTA) apexification are classical treatments for necrotic immature permanent teeth. The first tend to fail for lack of compliance given the high number of sessions needed; the second has technical difficulties such as material manipulation and overfilling. With both techniques, the root development is interrupted leaving the tooth with a fragile root structure, a poor crown-to-root ratio, periodontal breakdown, and high risk of fracture, compromising long-term prognosis of the tooth. New scientific literature has described a procedure that allows complete root development of these specific teeth. This regenerative endodontic procedure (REP) proposes the use of a combination of antimicrobials and irrigants, no canal walls instrumentation, induced apical bleeding to form a blood clot and a tight seal into the root canal to promote healing. MTA is the most used material to perform this seal, but updated guidelines advise the use of other bioactive endodontic cements that incorporate calcium and silicate in their compositions. They share most of their characteristics with MTA but claim to have fewer drawbacks with regards to manipulation and aesthetics. The purpose of the present article is to review pertinent literature and to describe the clinical procedures protocol with its variations, and their clinical application.

## 1. Introduction

**Figure 1:**
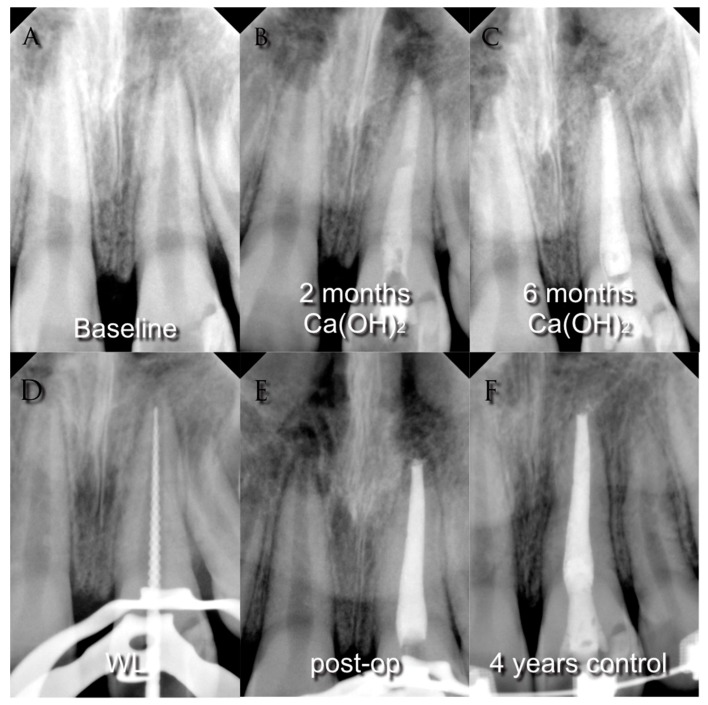
(**A**) Pre-operative radiograph of a young necrotic upper left central incisor with periapical lesion; (**B**) radiograph after two months medication with calcium hydroxide; (**C**) radiograph after six months medication with calcium hydroxide; (**D**) working length determination; (**E**) post-operative radiograph; (**F**) four-years control radiograph.

Since the 1960s, the procedure indicated to treat immature permanent teeth with loss of vitality was apexification [1,2], a technique that aims to obtain a calcified apical barrier that permits the canal to be filled in a conventional way afterward [3], see Figure 1.

This technique has been demonstrated to be predictable and successful; however, some complications remain [4]. The traditional apexification technique used calcium hydroxide, Ca(OH)_2_, a strong base with a high pH (approximately 12), that was originally used in endodontics as a direct pulp-capping agent in 1928 [5]. Ca(OH)_2_ is formed by a powder that when in contact with an aqueous fluid dissociates into calcium and hydroxyl ions. This reaction induces a hard-tissue deposition and high antimicrobial activity [6].

The reaction of periapical tissues to this material is similar to that of pulp tissue [7]. It produces superficial necrosis and subjacent mineralization due to the matrix production caused by low-grade irritation from the necrosis. Calcium ions are attracted to that collagenous matrix and initiate calcification [8]. The mineralization of an apical barrier is promoted by high pH and the absence of microorganisms. Calcium hydroxide has antibacterial properties: It releases hydroxyl ions that are highly oxidant and reactive and damage bacteria in different ways. The calcium ion instead, can stimulate enzyme pyrophosphatase, facilitating repair mechanisms [9]. This procedure consists in opening an access to the pulp, cleaning the canal using irrigation agents and manual files (generally slightly shorter to the apex), and applying a calcium hydroxide paste that is replaced periodically to promote a faster healing response; the first replacement is advised after 4–6 weeks, then every 2–3 months until the operator feels a barrier when probing the apex with an endodontic file. After this, it is advised to wait another 3 months to finalize the procedure [10]. After the mineralized barrier completion, the tooth canal is filled with gutta-percha and sealer [9]. Unfortunately, this procedure presents some disadvantages, such as being a long treatment, taking between 6 to 24 months to complete, where the patient needs to attend multiple times to assess progression and evaluate the need to change the medication. The advantages of changing the intra-canal dressing in between sessions are high pH maintenance, continuous delivery of OH^−^ ions to the periapical area, the possibility of renewing temporary cavity filling avoiding infiltrations, and to clinically assess the barrier formation. It also allows one to replace part of the dressing that has been washed out down the large apex, to maintain patience compliance, and to ensure complete contact between the calcium hydroxide and the apical tissues.

Not changing the intracanal medication may lead to the same result but at a later time and with a higher risk of infection [10]. Another disadvantage of this technique is the loss of mechanical strength which increases the risk of root fracture [11]. Several in vitro studies using immature permanent teeth of different animals [12,13,14,15] are in agreement with clinical observations of cervical fracture of calcium-hydroxide-treated teeth that have received minor impacts or none [11,16]. The flexural strength of dentin is given by links between hydroxyapatite crystals and collagen. The alkalinity of calcium hydroxide affects these links, weakening the dentin [12] and leaving it prone to fracture. Most of these studies have found non-significant damage when used for under a month followed by a continuous decrease of mechanical strength in time. One study did not find [17] significant changes in the resistance to mechanical stress, suggesting that it was probably because they used preparations with calcium hydroxide in low concentrations mixed with additives that could work as buffers and that the study was made on deciduous teeth, instead of immature permanent teeth.

**Figure 2:**
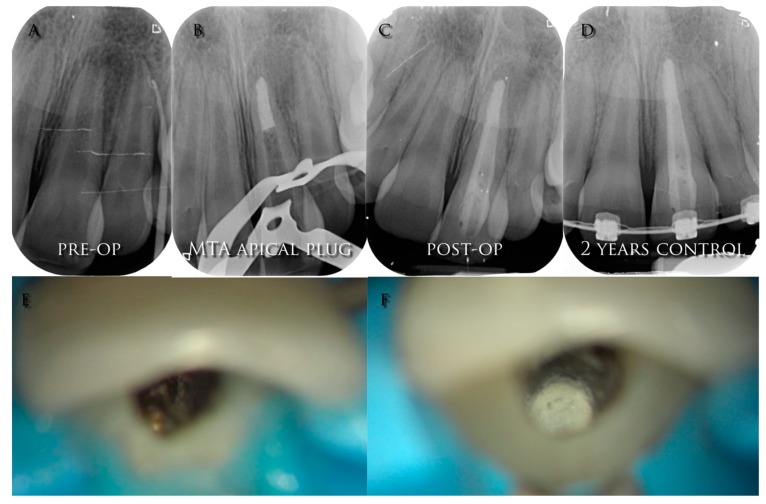
(**A**) Pre-operative radiograph of a young necrotic upper left central incisor with open apex and a periapical lesion; (**B**) radiograph of the MTA apical plug; (**C**) post-operative radiograph; (**D**) 2-years control radiograph; (**E**) intra-operative image of the open apex; (**F**) intra-operative image of the MTA apical plug.

More recently, another type of apexification named as “apical MTA plug” was described using a new material, Mineral Trioxide Aggregate (MTA), a biocompatible powder with fine hydrophilic particles that hardens in the presence of humidity [18], see Figure 2.

Hydration of the powder produces a colloidal gel with a pH of 12.5 that solidifies to a hard structure. The setting time for this material is around 4 h, its compressive strength at 21 days is around 70 MPa [19], and it presents a good sealing ability [20] and low cytotoxicity [21].

ProRoot MTA (Dentsply-Tulsa Dental Specialties, Johnson City, TN, USA) was the first commercial brand of MTA and probably the most widely used clinically since its introduction to the market in 1999 [22]. ProRoot MTA has two different versions: The original grey one, and since 2002, a white version. The chemical composition of grey ProRoot MTA is tricalcium silicate, dicalcium silicate, bismuth oxide, tricalcium aluminate, calcium sulphate dihydrate (gypsum), and calcium aluminoferrite. The white version does not have calcium aluminoferrite in its formula. They both have distilled water as the liquid for the mixture. Grey ProRoot MTA has an initial setting time of 70–74 min and a final setting time from 210 to 320 min [23]. White ProRoot MTA has been demonstrated to have faster initial and final setting times than grey ProRoot MTA [24].

Angelus MTA (Angelus, Londrina, Brazil) was the second commercial brand that has appeared on the market, which was then followed by numerous versions of different MTA, see Table 1. It has the following composition: Tricalcium silicate, dicalcium silicate, bismuth oxide, tricalcium aluminate, calcium oxide, aluminium oxide, silicon dioxide. It also uses distilled water as the liquid for the mixture. It presents an initial setting time of 8.5 ± 2.4 min; [23] however, another study reported an initial setting time of 34.03 ± 6.079 min and a final setting time of 41.57 ± 6.83 min [25].

The clinical procedures for the MTA apical plug technique comprise a first visit to access the canal, irrigate with NaOCl and place calcium hydroxide paste for one week. In the next session, the paste is rinsed, and the canal is dried with paper points. After mixing, MTA is applied with a carrier into the apical part of the canal and condensed lightly until a 3–4 mm plug is formed. A moist cotton pellet is then placed in the canal to promote setting of the coronal part of the material with humidity and the tooth is closed with a temporary restoration material. After 72 h, the MTA is set and a gutta-percha and sealer obturation can be made for the rest of the canal [19]. The suggested amount of MTA that needs to be inserted into the canal is such to obtain enough displacement resistance and to avoid leakage [26].

This technique offered an improvement in terms of timing, with only one appointment needed to perform it and another one to control setting of the material and fill the rest of the canal [27], with a positive success rate. While calcium hydroxide apexification and MTA Plug may have similar clinical and radiographic success rates and apical barrier formation rates, the fact that MTA was associated with a significantly shorter time to achieve apical barrier formation made a difference in the overall success assessment because many failures with calcium hydroxide were due to poor patient follow-up because of the extensive time of treatment [28]. This material is also reported as faster on apical hard tissue barrier formation and lamina dura formation on radiographs compared to calcium hydroxide [29]. Furthermore, MTA reduced the possibility of tooth fractures in the long-term in studies comparing it to calcium hydroxide [30,31].

MTA presents an antimicrobial effect against some microorganisms, such as facultative bacteria, but no effect on strict anaerobes species [32]. Biocompatibility assessments of a material include the general toxicity profile in a cell culture, implantation tests, and usage tests in experimental animals following accepted protocols. A comparative study has shown that MTA is more biocompatible than Super EBA (Reinforced zinc-oxide cement based on eugenol and ethoxy benzoic acid), IRM (Intermediate Restorative Material-Dentsply Caulk) and silver amalgam [33]. When MTA is applied directly to human tissues, it forms calcium hydroxide that releases calcium ions for cell attachment and proliferation [34], creates an antibacterial surrounding due to its alkaline pH [35], modulates cytokine output [36], stimulates differentiation and migration of cells that form hard tissue [37], and forms hydroxyapatite or carbonated apatite on the surface where it was applied, providing a biological seal [38,39,40].

From the list of drawbacks that have been mentioned for this material, such as tooth discoloration, retreatability difficulties, and overfilling, only the last one applies to the plug technique. In immature roots with either parallel or convergent walls, MTA could be placed in an ‘ideal’ position significantly more often compared to a divergent root canal [41]. To avoid this, the use of a barrier made of a collagen matrix [42,43] or a platelet-rich fibrin matrix [44] before applying the MTA plug serves as a resistance to prevent overfilling. A technique was also reported to control the MTA condensation in large apexes where small increases of MTA are applied and condensed with different sizes of Thermafil carriers [45].

Since the MTA apexification was first practiced and studied, several bioactive endodontic cements (BECs) became available on the market in the form of root repair materials, see Table 2, and endodontics sealers for root canal filling, see Table 3. It has also been claimed that these materials have properties similar to those of MTA but without its drawbacks. All these materials have different chemical compositions, mainly based on calcium and silicate, but they share one characteristic, the bioactivity [23]. In this case, bioactivity means the capacity of releasing Ca+ ions, electroconductivity, production of calcium hydroxide, formation of a layer between the cement and the dentinal wall, and formation of apatite crystals on top of the materials surface in a synthetic tissue fluid environment such as phosphate buffer saline [46,47,48].

Biodentine (Septodont, Saint-Maur-des-Fosses Cedex, France) presents the following composition: Tricalcium silicate, dicalcium silicate, calcium carbonate, zirconium oxide, calcium oxide, iron oxide. Liquid: calcium chloride, a hydrosoluble polymer, and water. The setting time is 6.5–45 min. The calcium-enriched mixture (CEM) cement (BioniqueDent, Tehran, Iran) ingredients are calcium oxide, silicon dioxide, Al_2_O_3_, MgO, SO_3_, P_2_O_5_, Na_2_O, Cl, and H&C. Liquid: Water-based solution; setting time is 50 min. EndoSequence, RRM, RRP (Brasseler, Savannah, GA, USA) has zirconium oxide, calcium silicates, tantalum oxide, calcium phosphate monobasic and filling and thickening agents in its composition, and the setting times of the putty version are: Initial 61.1 ± 2.5 min and final 208 ± 10 min [23].

Several studies have already validated the use of BECs as an alternative material in the MTA apical plug technique [49,50,51,52,53]. Unfortunately, the level of evidence on the placement of BECs instead of MTA for this technique is low, including only case reports and case series [30]. Similarly to the calcium hydroxide apexification, the apical plug technique using MTA or other BECs presents the limitations of an interrupted root development, leaving the tooth with a fragile root structure and a poor crown-to-root ratio leading to higher risk of root fracture [11,12,54,55,56,57,58].

An alternative endodontic procedure for the treatment of immature teeth that may promote further root development by attempting to regenerate the pulp-dentin complex was introduced by Nygaard-Ostby in 1961, who analyzed human and dog teeth [59]. He opened them, cleaned the pulp chamber, performed a pulpectomy, and flooded the canal with EDTAC (EDTA (ethylenediaminetetraacetic acid) with a quarternary ammonium compound); then, with a Hedstrom file, provoked bleeding, pushing it beyond the foramen and into the periapical bone. Then, the middle and coronal third of the canal was filled with a gutta-percha point sealed with chloro-percha paste. After performing histologic sections, he could appreciate that the blood clot was gradually replaced by granulation tissue, which in turn, was gradually transformed into fibrous connective tissue. The presence of granulation tissue was always followed by surrounding canal walls resorption, and this process was always succeeded by deposition of cementum as the granulation tissue became connective tissue. These tissues never really organized completely and did not fill the entire root canal. The author then advised performing other studies changing certain conditions such as make no injury to the periapical tissue or leaving apical pulp in cases of tooth vitality. Rule and Winter, in 1966, published the first case report of continued apical formation and apical closure on a necrotic immature mandibular premolar after root canal disinfection with instrumentation, polyantibiotic dressing, and absorbable iodoform placement followed by establishing bleeding into the canal system [60]. Nygaard-Ostby and Hjortdal also presented a case series in 1971, that had, as a result, ingrowth of fibrous connective tissue and sometimes cementum in mature teeth with vital pulp, while in necrotic cases no repair occurred. They recognized that the blood coagulum enhances the reparative processes and that, in order to achieve better healing, no instrumentation beyond the foramen is needed in vital cases [61]. Several other case reports, in addition to the above-mentioned, served as a starting point for all the actual pulpal regeneration protocols because they demonstrated that even in a necrotic tooth, a positive outcome could be obtained [62,63,64]. There was also the knowledge of cases of traumatically avulsed immature teeth that presented a necrotic but uninfected pulp and after re-implantation obtained revascularization [65,66] and of apical root formation on a traumatically intruded maxillary immature lateral incisor adding a collagen-calcium phosphate gel after root canal debridement [67].

The purpose of the present article is to review the published literature on the regenerative endodontic procedure (REP) and to present a step-by-step description of the clinical procedures for the revitalization of immature necrotic teeth, pointing out all the different variations in the protocol and their clinical application.

## 2. Terminology

Several terms have been used up to now to identify the topic treated in the present report.

Maturogenesis: The continued physiological root development [68]. Maturogenesis is more often used for describing root full development after direct pulp-capping [69].

Regeneration: The current American Association of Endodontists (AAE) “Glossary of Endodontic Terms” (2012) defines regenerative endodontics as “biologically-based procedures designed to physiologically replace damaged tooth structures, including dentin and root structures, as well as cells of the pulp-dentin complex” [70].

Revascularization: The re-establishment of the vascular supply to existing pulp in immature permanent teeth [66].

Revitalization: An ingrowth of tissue that may not be the same as the original lost tissue [71]. Authors who are skeptical about the nature of the new tissue capable of continuing root development because of its unpredictability use the word “revascularization”, arguing that the only certainty they have is the presence of blood supply [72]. This term has also been used in trauma publications for re-implantation of avulsed teeth or repositioning of luxated teeth with revascularization [73]. Its use for a different procedure may lead to confusion and the use of “induced or guided tissue generation and regeneration” has been advised instead [74]. Authors who use the term “revitalization” state that the new tissue within the pulp space, although not necessarily pulp, does not comprise only blood vessels. It comprises of vital cells that are required to lay down the new tissue. Therefore, the pulp space is filled with connective tissue of some type and this tissue is vital [75].

Geisler has proposed the definition “regenerative endodontic procedures” to unify all other terms previously described [76]. The AAE published a position statement [77] to inform all patients and clinicians that regenerative endodontics is a valid procedure for endodontists to perform. They also pointed out that the 2011-2012 ADA Current Dental Terminology included a new code (D3354) for pulpal regeneration within the endodontic section of the document, recognizing it as an endodontic procedure [78]. More recently, the European Society of Endodontology (ESE) has also published a position statement to affirm that revitalization procedures in immature teeth after pulp necrosis have become part of the endodontic treatment spectrum and should be considered as an alternative to apexification [79].

This conservative two-step procedure consists of the use of a combination of antimicrobials to reduce infection, no canal walls instrumentation, and induced apical bleeding to form a blood clot tightly sealed into the root canal in order to promote healing [80,81]. It can be performed in cases of pulp necrosis secondary to trauma [82], decay [83] or dental anomalies [84]. It is cost-effective, technically simple, can be executed with currently available resources, does not need expensive bio-technology [85], and eludes the chance of immune rejection and pathogen transmission from applying a tissue engineered construct in replace of the pulp [86]. With the right patient compliance, this procedure can lead to apical healing according to recent systematic reviews showing success rates of 91% with regenerative procedures. Secondary outcomes of increased root development (80%) and apical closure (76%) produced more variable results [87,88].

## 3. Clinical Step-By-Step for Endodontic Regenerative/Revitalization Procedures

**Figure 3:**
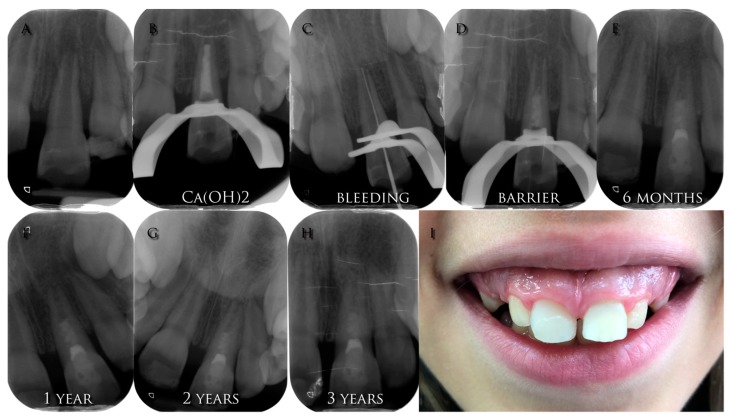
(**A**) Pre-operative radiograph of a young necrotic upper left central incisor with open apex and a periapical lesion; (**B**) radiograph of the calcium hydroxide medication; (**C**) working length radiograph of the file inducing bleeding; (**D**) radiograph of the coronal barrier positioned; (**E**) 6-months control radiograph; (**F**) 1-year control radiograph; (**G**) 2-years control radiograph; (**H**) 3-years control radiograph; (**I**) 3-years clinical image.

**Scheme 1:**
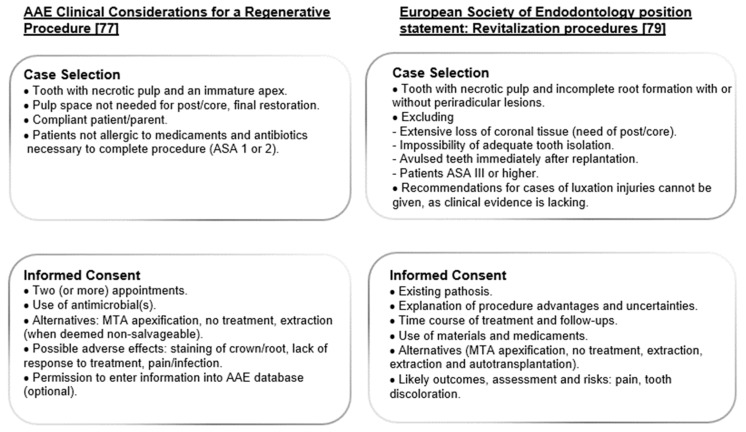

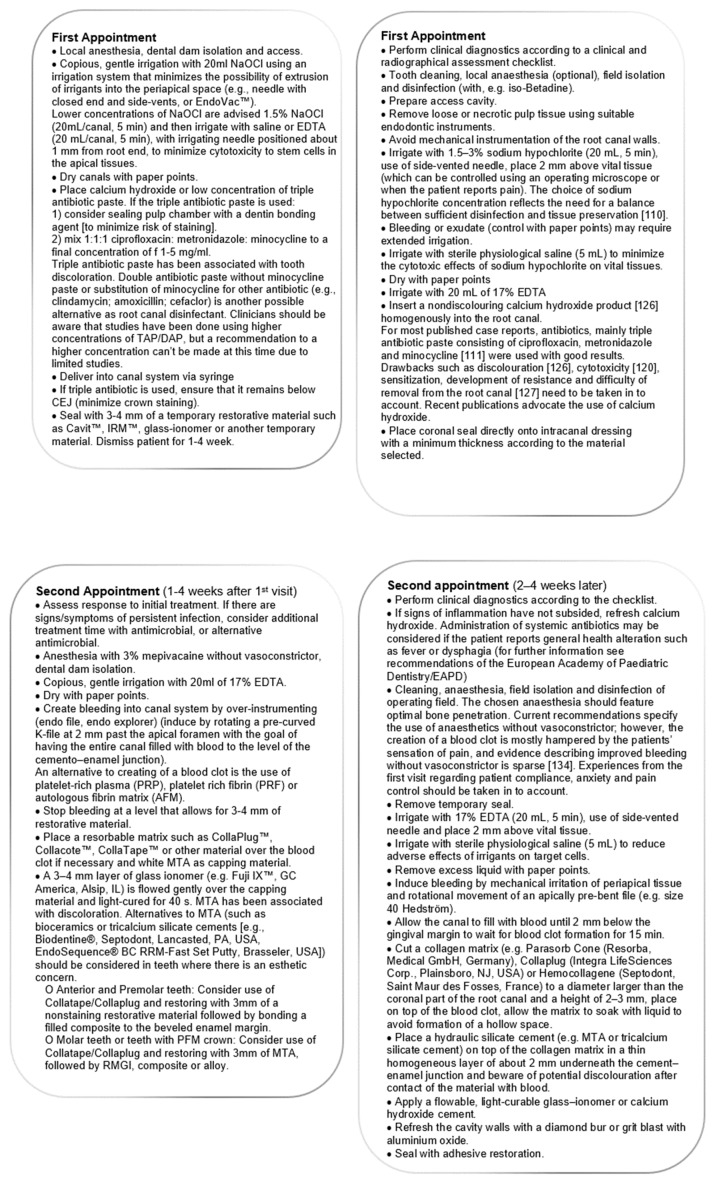

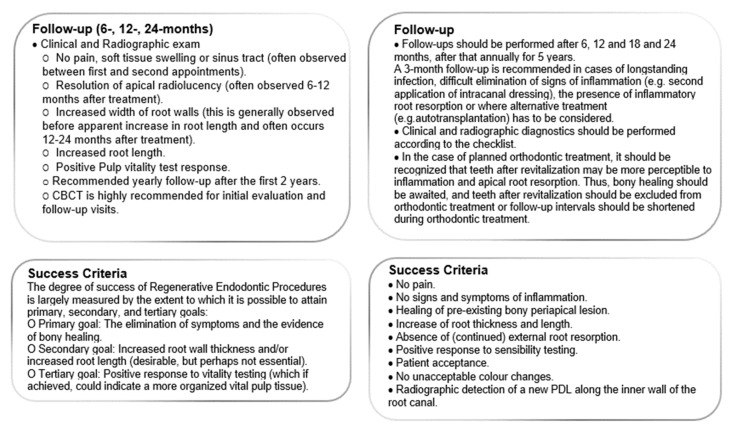
Comparison between American Association of Endodontists (AAE) (adapted from [77]) and European Society of Endodontology (ESE) protocols (adapted from [79]).

The AAE developed the “Clinical Considerations for a Regenerative Procedure”, as the guidelines to follow regarding this topic [77,89]. The ESE also published its position statement about revitalization procedures with clinical guidelines [79]. Both documents contain structured and evidence-based instructions for clinical procedures (Figure 3), which are summarized in the Scheme 1.

### 3.1. Case Selection

Case selection is of extreme importance as choosing a patient that does not correctly apply for this procedure may result in an unsuccessful outcome. Teeth with necrotic pulp and an immature apex, in which the pulp space is not needed for a post/core build-up, are candidates. The patient must have high compliance since multiple appointments are required. Case reports generally describe very young healthy patients (6–18 years old), with good regenerative potential, greater capability to promote healing, and a higher amount of stem cells available [90,91]

The probability of success in teeth with immature apices might depend on how large the apical opening is: The larger it is, the more possible the ingrowth of vessels and stem cells into the root canal is [92]. It also seems that apices larger than 1 mm are more likely to be revascularizated [93]. Not only is it accepted that short and open roots are more conducive to the ingrowth of tissues, but it has also been suggested that a short and open apex may indicate the increased presence and number of stem cells of the apical papilla (SCAP) [94].

A further pre-operative consideration is represented by the duration of the infection. Hypothetically, the longer the pulp infection lasts in immature teeth, the lower the chance to find survived pulp tissue or stem cells remaining. Additionally, longer infection renders the disinfection more difficult to accomplish [95].

### 3.2. Informed Consent

This document must include all the informative material needed to make an informed decision. It must explain that the treatment will take place in two or more appointments with frequent need of follow-ups, the allergenic potential of the antimicrobials used and other adverse reactions such as tooth discoloration, non-healing, pain and infection. Informed consent must also explain other viable alternative treatments such as apexification, extraction, or no treatment at all. If clinicians want to include a case report to the AAE regenerative database, they should also obtain the parent/guardian permission for that matter.

### 3.3. First Appointment

Local anesthesia is used, a rubber dam is mounted, and cameral access are performed on all cases.

At this point, if a little resistance when inserting a file is felt or the patient reports pain, the presence of residual vital tissues should be hypothesized and, according to Jung et al., an apexogenesis procedure should be performed [96].

#### 3.3.1. Irrigation

After minimal or no instrumentation, the irrigation must be copious and gentle, minimizing the possibility of extrusion into the periapical space. Sodium hypochlorite (NaOCl) is the most used irrigating solution in concentrations ranging from 1% to 6%. Some authors have had experimental results that suggest that viability of the existing cell population is maintained when using lower concentrations of NaOCl, leading to an increase of success in treatment outcome [97] and that using them would prevent further damage in cases of inadvertent extrusion beyond apical foramen [98].

In a later study, Trevino et al. found that the survival rate of stem cells of the apical papilla (SCAP) exposed to 6% NaOCl, 17% EDTA, and then 6% NaOCl once more, was 74% [99]. Thus, it has been suggested that NaOCl in a concentration closer to 6%, if used, associated to 17% EDTA, may partially reverse the harmful effect on stem cells, promoting survival and differentiation of stem cells of the apical papilla [100].

NaOCl should always be slowly extruded from the syringe to reduce the risk of periapical introduction [46,101]. A needle with closed end and side-vents [102] or an EndoVac (Discus Dental, Culver City, CA, USA) are advised to reduce possible extrusion of the liquid [103]. Furthermore, the needle should be moved slowly up and down and maintained 2 mm short of the apical foramen [56]; this is based on the knowledge that when a syringe plunger pushes the solution slowly, it only extends 1 mm beyond the tip of the needle [104].

A lack of adherence to the surface of the root canal by stem cells has also been described when using NaOCl, thus recommending the use of a saline solution to wash out all the NaOCl solution [105]. One study also described a single appointment technique using only 6% NaOCl and 2% chlorhexidine (CHX) as coronal irrigation [106]. Chlorhexidine has been shown to be cytotoxic to stem cells [93] and to create a precipitate in combination with NaOCl [107]. To prevent this reaction, the use of pure alcohol, distilled water, or saline solution between applications is needed [108]. This effect can also be reversed by using l-α-lecithin that completely neutralizes cytotoxicity after the application of CHX [109,110]. NaOCl, in combination with hydrogen peroxide, has also been described [111].

Current protocols advise the use of NaOCl in a percentage of 1.5% [89] or 1.5–3% [79], gaining a balance between disinfection and cell protection [112].

#### 3.3.2. Antimicrobial Medication

The most used intracanal antibiotic dressing is the tri-antibiotic paste (TAP), introduced by Hoshino et al. as an effective solution to eradicate bacteria from the dentin of the infected root and promote healing of the apical tissues. It is a mixture of equal parts of ciprofloxacin, metronidazole, and minocycline with sterile saline to form a paste-like consistency mixture [113]. Some authors also describe an adaptation for the TAP with ciprofloxacin 200 mg, metronidazole 500 mg, minocycline 100 mg, and a carrier that could be macrogol ointment or propylene glycol [114]. This paste can be inserted into the canal 1–2 mm short of apex, with a lentulo spiral or a syringe-type carrier and then tapped down with a moist cotton pellet. The capacity of minocycline to discolor the tooth is well known [115]. If using TAP, the pulp chamber could be sealed with a dentin bonding agent and/or flowable composite to avoid this unwanted effect [116,117]. Another adaptation is to exchange minocycline for cefaclor [118], or if esthetic is crucial, it is also possible to eliminate minocycline from the mix using a double antibiotic paste (DAP) [119,120].

Even if studies have identified TAP as biocompatible [121], an undiluted mixture of antibiotics has a detrimental effect on stem cell survival and titrating the proper concentration seems to be important according to newer studies. The concentrations must be bactericidal, but with minimal damaging effects on stem cell viability [122]. Updated content from the AAE clinical considerations for regenerative procedures advises the use of a low concentration of TAP of 1–5 mg/mL [89].

Another medication that is extensively used is calcium hydroxide, Ca(OH)_2_, a root canal disinfectant and hard tissue repair stimulator [6]. When there is sensitivity to one of the antibiotics of TAP or DAP, its use was advised [56]. Given its high pH, this medication can destroy cells from the apical papilla and periapical tissues that are fundamental for the repair process [84,123], could be conducive to uncontrolled calcification of the canal space, preventing the ingrowth of soft tissue with an odontogenic potential and may limit the possibility to induce bleeding on the second visit. This is the reason why Ca(OH)_2_ must be applied only in the coronal half of the root canal, to permit a positive outcome [95]. It must not be applied with a lentulo spiral, but with a syringe-type carrier instead and tamped down gently with a moist cotton pellet to the junction of the coronal and middle third of the root length, thus obtaining the beneficial properties and limiting the possible toxicity [56]. Cases treated with Ca(OH)_2_ also displayed intra-canal calcifications that may interfere with the continued thickening of the dentinal walls of these immature teeth [124].

On the other hand, calcium hydroxide has been proven to be effective in regenerative procedures [114,125,126,127]. In fact, recent publications advocate the use of calcium hydroxide to avoid TAP drawbacks, such as discoloration [128], cytotoxicity [122], sensitization, development of resistance, and the complications for its removal from the root canal [129].

Formocresol has also been described as an intra-canal medication used in regenerative procedures [130].

Studies are still not clear about how much time the medication needs to act inside the canal to reach its aim [131]. Many articles do not mention this information and, when mentioned, the range of use is highly variable, describing periods from 2 [132] to 11 weeks [133], with periods of 2 to 4 weeks [76,131] or 3 weeks [83,134,135] being the more advised. In a review, assessing the reported clinical success of procedures made with TAP or calcium hydroxide, it seems that 2 to 4 weeks may be a sufficient period [131].

#### 3.3.3. Coronal Seal

After the application of the antimicrobial paste, 3–4 mm of interappointment material, such as Cavit (3M ESPE, St. Paul, MN, USA) should be applied onto the canal and a tighter coronal filling should be performed, i.e., using glass-ionomer cements [56,76,95,134]. Other studies have only used Cavit or IRM (Dentsply/Caulk, York, PA, USA) alone [57,83,131,132,133,135].

### 3.4. Second Appointment

If the patient has persistent symptoms and/or there are signs of infection, additional treatment time with the same antimicrobial or a different one may be necessary [96]. If there are no symptoms or infection, it is appropriate to continue with the next phase of the regenerative procedure [76].

Local anesthetic without a vasoconstrictor is used in most of the cases, in order to permit the induced bleeding [136].

#### 3.4.1. Irrigation

After dam isolation and removal of the temporary restoration, the canal must be irrigated to remove the medication. As previously reported, NaOCl and CHX may be cytotoxic to the stem cells and may inhibit their ability to adhere to dentin, thus the use of a sterile saline solution could be more advisable in this phase [93,105].

Newer articles are describing the advantages of irrigating with 17% EDTA at this point of the procedure [137]. EDTA releases and exposes growth factors from dentin [138,139,140] and being a chelating agent, it can decalcify the surface of the root canal and expose the collagen fibers in dentin [140]. Cell adhesion motifs in collagen could promote the attachment of new cells, while the decalcification of the dentin releases bound growth factors that can attract new cells and stimulate their differentiation into cells with odontoblast-like properties [70,140].

#### 3.4.2. Promoting Blood Clot

Apical tissues beyond the confines of the root canal need to be stimulated with a sterile endodontic file or explorer to induce bleeding into the canal space [131]. It takes approximately 15 min to allow the blood to clot and stabilize below the cementoenamel junction, applying intracanal pressure with a sterile cotton pellet soaked in a sterile saline solution [56]. If bleeding does not occur, it may be necessary to dip a file with a small bend in 17% EDTA and over instrument to prevent coagulation of the blood [131]. If bleeding still does not occur, in a tooth with more than one root, it is possible to draw with a syringe, blood from the adjacent canals and put it in the dry ones [83]. If it is not, an alternative is to draw blood with a venipuncture and inject it into the canal system [76]. If none of this is achieved there are also cases describing the injection of platelet-rich plasma (PRP) [141] or autologous fibrin matrix (AFM) into the canal as a solution [76]. They contain growth factors, that along with other beneficial actions, induce cell differentiation, initiate vascular ingrowth, and improve wound healing [142,143]. Currently, there is no evidence showing that a blood clot is required for the formation of repaired tissues in the canal space [57]. One study that used a dog model showed no statistically significant difference in healing by inducing a blood clot into the canal, although the authors suggested the inclusion of a blood clot to improve the chance of healing [118]. Nevertheless, other articles have reported that the stimulation of a blood clot is essential to promote healing, working as a scaffold [80], and that this evoked-bleeding step in regenerative endodontic procedures leads to a significant increase in the expression of undifferentiated mesenchymal stem cell markers in the root canal space [144].

At this moment, the use of a barrier over the pulp space to facilitate the application and compaction of the material in order to obtain a tight seal has been advocated. Collagen wound dressings in a tape and cylindrical shape may help to prevent overextension of sealing materials into the canal [136].

#### 3.4.3. Sealing

The guidelines advise the use of a bacteria-tight sealing material on top of the barrier to obtain the desired sealing. It can be a hydraulic silicate cement, white MTA, a new bioactive cement, or grey MTA in cases where aesthetics is not an issue. This should always be applied 2–3 mm underneath the cement-enamel junction [79,89].

Grey MTA has been shown to provide an effective bacteria-tight seal [145] and, lamentably, some cases of tooth discoloration [46]. This has also been encountered with the use of white MTA, but in very rare cases and over time (12 months from application) [128,146,147]. A barrier of 3-4 mm of MTA should be sufficient to obtain the desired seal [131]. To overcome the discoloration problem, MTA can be put only under the CEJ (Cemento-enamel junction) or another material can be used [89]. As described previously, to overcome this and other drawbacks, the bioactive endodontic cements (BECS) represent alternative materials that also have the regenerative procedures as a clinical indication and have been used in cases of necrotic immature permanent teeth [30].

BECs induce hard tissue formation and are biocompatible, non-toxic, non-resorbable, and unaffected by blood contamination [23].

An in vitro study on the effect of BEC materials on proliferation and differentiation of odontoblasts for regenerative endodontic treatments reported that MTA and tricalcium-silicate-based materials induced proliferation of stem cells from the apical papilla, but Biodentine (Septodont, Saint-Maur-des-Fossés, France) also showed differentiation of these cells [148]. Thus, Biodentine has been recommended for placement on top of the blood clot as a sealing material [149]. This cement has been reported to be biocompatible, bioactive, and to induce the osteogenic differentiation and mineralization of dental pulp and mesenchymal stem cells [150]. It sets in approximately 12 min [151], does not wash out easily, and is easy to handle [152]. In the composition of this cement, there is only synthetic tricalcium silicate, purer than when the natural product is purified [153], it does not present heavy metals [154] and its color is maintained with time [155]. Its tricalcium silicate particles are smaller than those from MTA [153] and the physical properties of Biodentine such as flexural strength, Vickers hardness, and elastic modulus are higher than those of MTA but similar to dentine [156].

Endosequence root repair material (Brasseler, Savannah, GA, USA) is a premixed bioactive cement produced as a ready-to-use syringeable paste or compactable putty, with easier manipulation over MTA [157]. The moisture present in the dentinal tubules seems to be sufficient for its setting [151], even if contradictory results have been obtained in laboratory settings about solubility and longer setting times [158]. In a study regarding this material [159], focused on its relation with different fluids, authors claim that in vivo, environmental conditions such as variable moisture or the presence of blood may alter its function and that in vitro studies reported that this material forms hydroxyapatite in the absence of carbon dioxide that is actually present clinically, leading to a calcium carbonate formation instead. When set, it creates a mechanical bond with dentin that provides dimensional stability [160]. In in vitro studies, it has been demonstrated to be comparable to MTA in marginal adaptation and thus sealing ability [161,162], with a biocompatibility similar to MTA [163,164].

EndoSequence Bioceramic Putty (Brasseler, Savannah, GA, USA) has been used indistinctly with MTA to assess success on regenerative procedures [165].

A calcium-enriched mixture (CEM) has been proposed as a valid alternative to MTA for regenerative endodontic procedures [83,166]. CEM is a tooth-colored, water-based cement. Its powder comprises various concentrations of calcium salt, calcium oxide, calcium silicate, and calcium phosphate, mixed with a water-based solution. The surface characteristics of set CEM are similar to human dentin, promoting hydroxyapatite formation and might also promote differentiation in stem cells and induce hard tissue formation [167,168].

In the case of bioactive materials, most of the cases describe the application of a temporary cement to await the final setting time before performing the definitive filling [165,166]. After application of MTA, some authors put a temporary cement over MTA for one day to assure its setting [135], others instead wait an hour with a moist cotton pellet over MTA to leave it to set in the same appointment [133] and then performed the definitive restoration, generally using a glass-ionomer base and an acid-etched composite resin. An in vitro study assessing the bond strength of the filling placed immediately after the application of MTA and BECS, advises a delayed definitive filling for MTA and the possibility of same-session filling only for Biodentine (Septodont, Saint-Maur-des-Fossés, France), waiting only 12 min between the cement application and the composite restoration [169].

When using MTA on regenerative procedures in animal studies, it provided a greater success rate for increasing root width, length, and healing compared to previous techniques and traditional materials [170,171,172,173,174,175,176]. In human studies, ProRoot MTA grey [177,178] ProRoot MTA white [136,179], Angelus MTA grey [177,180], Angelus MTA white [75], Endosequence Bioceramic Putty [163], Biodentine [149,181], and CEM cement [83] have been used as a coronal plug for regenerative procedures successfully. Nonetheless, more than 85% of the related studies used MTA as the blood clot sealer [182].

### 3.5. Follow Up

Periodic appointments for clinical and radiographic evaluation should be scheduled. Some authors suggested follow-ups every 3 months at the beginning [133], others suggested 3-, 6-, 12-, and 18-month recalls [83]. Most clinicians suggest that during the first year, 3-month recalls should be programmed, followed by 6-month recalls unless clinical symptoms develop [56].

Clinical examination should show no pain to palpation/percussion and no soft-tissue swelling or sinus tract. Periapical radiographs should be made to observe resolution of an apical radiolucency (if present before treatment), an increased width of root walls, and/or an increased root length. Chueh et al. [124,183] reported referential timings for these findings to occur: Apical radiolucencies resolution within 3–21 months (mean 8 months) and “nearly normal” root development in 10 to 29 months (mean 16 months) after a regenerative endodontic procedure protocol using calcium hydroxide.

A pulp vitality test must also be performed since some cases have described a positive response after the treatment [84,119,124,133].

If signs and symptoms persist, or after two years, no radiographic evidence of healing and root development is found, this indicates a failure of the procedure and another treatment such as calcium hydroxide or MTA apexification should be performed [76]. Other authors advise more radically that, if within 3 months no signs of regeneration are found, methods that are more traditional can be initiated [72].

### 3.6. Success Criteria

The degree of success for this protocol is measured by the extent to which it is possible to attain the primary, secondary, and tertiary goals; the primary goal is the elimination of symptoms and evidence of bone healing. The secondary goal (that is desirable but not essential) includes root maturation, while the tertiary goal (that indicate the highest level of success) is the positive response to vitality testing [89].

## 4. Discussion

Even if long-term outcome studies in humans do not yet exist [87], a recent systematic review and meta-analysis reported that revitalization and MTA apexification had high survival rates and positive outcomes with no significant differences between each other [87].

A retrospective study comparing the outcome of revascularization to MTA apexification and calcium hydroxide apexification reported a survival rate (defined as retention of the tooth in the arch at the time of the post-operative recall) of 100% for revascularization at an average of 14 months after treatment. This compared favorably with the 95% survival rate for the MTA apical plug apexification and the 77% survival rate for the apexification obtained using calcium hydroxide. Furthermore, the revascularization group produced a significantly higher percentage increase in root width (28.2%) compared to the MTA apexification group (0%) or calcium hydroxide apexification (1.52%) and a significantly higher percentage in the root length measurements (14.9%) compared to MTA apexification (6.1%) and calcium hydroxide apexification (0.4%) [55]. The hard tissue barrier formed with the conventional calcium hydroxide apexification technique has been described as “swiss-cheese-like”, because of the many soft tissue inclusions, thus representing a very permeable and weak barrier and more attention is needed when filling the root canal with gutta-percha and sealer [184].

A radiographic analysis, considering success as the regression of signs and symptoms associated with infected necrotic teeth as well as radiographic evidence of continued root development, showed that there was evidence of continued root development in all the groups tested. Regenerative procedures protocols changed only for the intra-canal medication used, TAP, Ca(OH)_2_, and formocresol, comparing them to controls (MTA apexification or regular root canal treatment). The TAP group showed the highest increase of dentin wall thickness compared to the other groups. In the Ca(OH)_2_ group, the authors measured root wall thickness at different heights because they noticed a difference. In cases where the dressing was applied on the coronal half of the root, the percentage increase of the dentinal wall was 53.8%, and when it was placed beyond that point the increase of the root wall thickness was only of 3.3%. The formocresol group showed the lowest increase in dentin wall thickness [125].

Despite the fact that it is well accepted that revitalization procedures heal apical periodontitis using MTA as a coronal barrier [182], some unsuccessful outcomes have also been described with this clinical protocol, reporting absence of an increase in root length [185], in root wall thickness [136,178], and in the formation of the root apex [178]. These results have been reported to probably be a consequence of a tooth history characterized by a long-time trauma, that compromised the vitality of the Hertwig epithelial root sheath [178].

The lack of response to a pulp vitality test does not necessarily indicate a lack of vitality, as many cases with radiographic proof of root maturation, which should indicate the presence of vital tissue in the canal space, responded negatively to vitality tests [186]. Even though this new protocol can be challenging and a successful outcome is not always guaranteed, it represents an improvement over previous techniques since the possibility of thickening or raising the length of the immature root is present [56].

Although the lack of a real regeneration with substitution of desired tissues has been observed and there is no evidence about what kind of tissues are obtained after REPs, histologic evaluation of tissues obtained after regenerative procedures in one animal study on dog immature necrotic teeth revealed different real tissue-like cells, showing that three types of tissues were generated after treatment, including cementum-like tissue along the dentinal walls responsible for root wall thickening, bone-like tissue, and periodontal ligament-like tissue [71]. In addition, in one case, partially survived pulp tissue and the existence of odontoblast cells lining the dentinal walls were evident. The study concluded that the tissue formed in the canal space is not pulp and does not have the function of the pulp tissue, which means that revascularization is not pulp regeneration but resembles the wound repair process. Another study suggested that there was approximately a 30% chance of pulp tissue re-entering the pulp space [187]. The dental papilla at the apex contains stem cells called SCAP that have been recently described as very robust [188] as they may survive infections and permit root maturation, while the survived dental pulp stem cells (DPSC) may rebuild the lost pulp tissue in the canal and give rise to replacement odontoblasts for substituting the damaged primary ones [90]. Whether the new vital tissue is truly pulp or pulp-like it does not affect the result, if there is healing and continued development of the root canal walls and apex [56,84].

For regeneration to happen in the dentin pulp complex, three factors are needed: A permissive environment in which the regeneration can occur, cells capable of secreting extracellular matrices of the dentin pulp complex, and secretion of molecular signals and interactions between cells for upregulation of cellular synthesis and secretion of new tissue [70,189]. The adequate environment in this clinical procedure is the evoked bleeding that becomes a blood clot that serves as a scaffold. In newer studies, platelet-rich plasma or platelet-rich fibrin are used to have a more predictable result [92]. In a study comparing MTA apexification and regenerative endodontic procedures using three different scaffolds (blood clot, platelet-rich plasma + collagen, and platelet-rich fibrin matrix), there was no apical closure, root lengthening or dentinal wall thickening for the MTA apical plug apexification. The blood clot used as a scaffold presented 60% periapical healing, 66.67% good apical closure, 40% good root lengthening, and good results of dentinal wall thickening in 50% of cases. In the case of platelet-rich plasma + collagen, 80% apical healing, 60% good apical closure, 40% good root lengthening, and 20% dentinal wall thickening. For the platelet-rich fibrin matrix, values were 98% excellent periapical healing, 40% good apical closure, 99% excellent root lengthening, and 60% excellent dentinal wall thickening [190]. A new population of mesenchymal stem cells residing in the apical papilla of permanent immature teeth has been discovered; named stem cells from the apical papilla (SCAP), they appear to be the source of the cells that are responsible for the continued root formation. Conservation of these cells when treating immature teeth with regenerative procedures may allow continuous formation of the root to its completion, even if we slightly over-instrument to induce bleeding, SCAP and periodontal cells would migrate into the intra-pulpal space forming new tissues [191]. SCAP cells have been proven to maintain survival and differentiation potential after infection [192,193].

Growth factors and other molecules with chemotactic properties are involved in cell recruitment and cell proliferation. Epigenetic modifiers, medicaments, and materials such as EDTA can release and expose these bioactive molecules in dentin to influence stem cell behavior [194].

The clinical procedures described in the present manuscript may also have some drawbacks, including possible tooth discoloration from minocycline [116], grey MTA [117], white MTA [136], and increase of the time of the treatment in the case of maintained clinical signs of inflammation or difficulties to disinfect the root canal space (compared with a MTA apical plug performed in one visit) [84]. Another difficulty is the impossibility to induce bleeding, described in different studies, even with the use of anesthetics without a vasoconstrictor [83,143,186]. In a case series of molars medicated with calcium hydroxide, the operators found that in mesial canals of inferior molars and buccal canals of the superior molar it was impossible to achieve an adequate level of hemorrhage, so they draw some from the palatal (superior molars) or distal (inferior molars) canal to supply for the need in the others [186]. Root canal calcification/obliteration, mostly using Ca(OH)_2_ as an antimicrobial in this procedure, was another reported drawback [124,178,183].

## 5. Conclusions

The outcome of classical conservative procedures to treat immature permanent teeth with pulp necrosis is healing, but with a higher risk of fracture compared with the fully developed teeth. Shifting apexification for REPs is clinically positive for patients. The successful outcome for this new REPs procedure depends mainly on canal disinfection, the placement of a matrix in the canal for tissue ingrowth (scaffold), and a bacterial-tight seal of the access opening. Pulp stem cells artificially applied onto the root canal are very far from a clinical reality, that is why we need to rely on cell homing, learning the release strategies of signaling molecules.

In future, a bigger distinction between infected necrotic pulp and irreversible pulpitis on immature permanent teeth should be made since the microenvironment in the canal space and status of apical papilla and Hertwig’s epithelial root sheath is different. For irreversible pulpitis cases, partial resection of the pulp and regrowth with a scaffold would be a possibility [184].

Predictable disinfection protocols and easily operated, non-discoloring scaffold sealing materials are needed.

Future research should concentrate on demonstrating histological changes in human teeth treated with this procedure and creating new techniques in the tissue engineering field that could increase the chance of achieving a real regeneration of the pulp-dentin complex. Once laboratory procedures are perfected, a new clinical procedure can be proposed, ideally using well-known cells, scaffolds such as PRP, and provoking the release of the right signaling molecules. The idea of keeping this technique clinical and achievable would help make it available for all patients.

## Figures and Tables

**Table 1:** MTA materials commercially available.

Name	Manufacturer	Composition	Setting Time
ProRoot Mineral trioxide aggregate (Grey)	Dentsply Tulsa Dental Specialties, Johnson City, TN, USA	Tricalcium silicate, dicalcium silicate, bismuth oxide, tricalcium aluminate, calcium sulphate dihydrate (gypsum) and calcium aluminoferrite Liquid: distilled water	Initial setting time has been reported from 70 to 74 min, whilst the final setting time is 210–320 min
Tooth-coloured ProRoot Mineral trioxide aggregate (White)	Dentsply Tulsa Dental Specialties, Johnson City, TN, USA	Tricalcium silicate, dicalcium silicate, bismuth oxide, tricalcium aluminate, calcium sulphate dihydrate or gypsum Liquid: distilled water	4 h
Angelus MTA (Grey and White)	Angelus, Londrina, Brazil	Tricalcium silicate, dicalcium silicate, bismuth oxide, tricalcium aluminate, calcium oxide, aluminium oxide, silicon dioxide Liquid: distilled water	The initial setting time of White Angelus MTA has been reported to be about 8.5 ± 2.4 min; however, other studies reported 130–230 min as the setting time for Angelus MTA
PD MTA White	Produits Dentaires SA, Vevey, Switzerland	SiO_2_, K_2_O, Al_2_O_3_, Na_2_O, Fe_2_O_3_, SO_3_, CaO, Bi_2_O_3_, MgO. Insoluble residues of CaO, KSO_4_, NaSO_4_ and crystalline silica. To mix with distilled water.	The material starts setting after approximately 10 min and the final setting time is 15 min. It is not necessary to wait for the final setting to continue the treatment procedure.
Endocem MTA	Maruchi, Wonju, Korea	CaO, Al_2_O_3_, SiO_2_, MgO, Fe_2_O_3_, SO_3_, TiO_2_, H_2_O/CO_2_, bismuth oxide	4.5–15 min
MicroMega MTA	MicroMega, Besancon, France	Tricalcium silicate, dicalcium silicate, tricalcium aluminate, bismuth oxide, calcium sulphate dehydrate and magnesium oxide	The manufacturer has claimed that the MicroMega MTA setting time is 20 min; however, there are reports that announced MM MTA has a setting time of 120–150 min
MTA Bio	Angelus; Londrina, or Angelus Solucoes Odontologicas, PR, Brazil)	Portland cement and bismuth oxide	The initial setting time of MTA Bio is 11 min. The final setting time of the material is 23.22 min
MTA Plus (White)	Avalon Biomed Inc., Bradenton, FL, USA	Tricalcium silicate, 2CaOSiO_2_, Bi_2_O_3_, 3CaOAl_2_O_3_ and CaSO_4_	MTA Plus setting time is 128 ± 8 min. In contact with moisture the material needs longer time to set
MTA Plus (Grey)	Avalon Biomed Inc., Bradenton, FL, USA	Tricalcium silicate, dicalcium silicate, bismuth oxide, tricalcium aluminium oxide, calcium sulphate and Ca_2_(Al,Fe)_2_O_5_	Initial Setting Time at 37 °C: ~15 min when thickly mixed with gel; otherwise longer for sealer (~3 h.)
OrthoMTA	BioMTA, Seoul, Korea	Tricalcium silicate, dicalcium silicate, tricalcium aluminate, tetracalcium aluminoferrite, free calcium oxide and bismuth oxide	324.0 ± 2.1 min
RetroMTA	BioMTA, Seoul, Korea	Calcium carbonate, silicon oxide, aluminium oxide and hydraulic calcium zirconia complex; Liquid: water	Initial setting time of 150–180 s and final setting time of 360 min
Aureoseal MTA	Giovanni Ogna and Figli, Muggio, Milano, Italy	The powder consists of Portland cement, bismuth oxide, setting-time controllers, plastifying agents and radiopaque substances. The liquid is distilled water	No setting time has been reported for the material
CPM MTA	EGEO SRL, Buenos Aires, Argentina	MTA, calcium chloride, calcium carbonate, sodium citrate, propylene glycol alginate and propylene glycol	The initial setting time of End-CPM is 6–15 min, whilst the material’s final setting time is 22–27 min

**Table 2:** Bioactive endodontic cements for root repair.

Name	Manufacturer	Composition	Setting Time
BioAggregate	Innovative BioCeramix, Vancouver, BC, Canada	Tricalcium silicate, dicalcium silicate, calcium phosphate monobasic, amorphous silicon oxide and tantalum pentoxides Liquid: deionized water	Based on the manufacturer data sheet, BioAggregate has a setting time of 240 min
Biodentine	Septodont, Saint-Maur-desFosses Cedex, France	Tricalcium silicate, dicalcium silicate, calcium carbonate, zirconium oxide, calcium oxide, iron oxide Liquid: calcium chloride, a hydrosoluble polymer and water	The setting time of Biodentine has been reported as 6.5–45 min
Calcium-enriched mixture (CEM) cement	BioniqueDent, Tehran, Iran	Calcium oxide, silicon dioxide, Al_2_O_3_, MgO, SO_3_, P_2_O_5_, Na_2_O, Cl and H&C Liquid: water-based solution	50 min
EndoBinder	Binderware, Sao Carlos, Brazil	Al_2_O_3_ and CaO	60 min
Endocem Zr	Maruchi, Wonju, Korea	Calcium oxide, silicon dioxide, aluminium oxide, magnesium oxide, ferrous oxide, zirconium oxide	-
EndoSequence, RRM, RRP	Brasseler, Savannah, GA, USA	Zirconium oxide, calcium silicates, tantalum oxide, calcium phosphate monobasic and filling and thickening agents	The setting time of EndoSequence putty is 61.1 ± 2.5 min and the final setting time is 208 ± 10 min
NeoMTA Plus	Avalon Biomed Inc., Bradenton, FL, USA	Tricalcium silicate, dicalcium silicate, tantalite, calcium sulphate and silica	NeoMTA Plus has had a 50- to 60-min setting time when prepared with putty consistency; otherwise, when used as a root canal sealer with loose consistency, it may take 5 h to set
Quick-Set	Avalon Biomed Inc., Bradenton, FL, USA, patent pending	Monocalcium aluminate powder that contains bismuth oxide (as a radiopacifier) and hydroxyapatite	12 min
iRoot FS (Fast setting), iRoot BP (Injectable) and iRoot BP Plus (Putty)	Innovative BioCeramix Inc., Vancouver, Canada	iRoot FS: calcium silicates, zirconium oxide, tantalum oxide and calcium phosphate monobasic iRoot BP (BioCeramix Inc.) and EndoSequence BC sealer (Brasseler USA) have had the same formula including zirconium oxide, calcium silicates, tantalum oxide, calcium phosphate monobasic, and filler and thickening agents	iRoot FS showed setting after 1 h, iRoot BP and iRoot BP Plus became solid after 5–7 days
Tech Biosealer Capping, Tech Biosealer Root End, Tech Biosealer Apex	Isasan, Como, Italy	Mixture of white CEM, calcium sulphate, calcium chloride, bismuth oxide, montmorillonite	The final setting time of various types of Tech Biosealer differ from each other. Tech Biosealer Capping has a final setting time of 55 min.

**Table 3:** Bioactive Endodontic Cements for root canal filling.

Name	Manufacturer	Composition	Setting Time
BioRoot RCS (Root canal sealer)	Septodont, Saint-Maur-desFosses Cedex, France	Tricalcium silicate, zirconium oxide (opacifier) and excipients in its powder form, and calcium chloride and excipients as an aqueous liquid	Less than 4 h
Endosequence BC (Bioceramic) Sealer	Brasseler, Savannah, GA, USA	Zirconium oxide, calcium silicates, calcium phosphate monobasic, calcium hydroxide, filler and thickening agents.	Setting time is 4 h measured according to ISO 6876:2001. However, in very dry root canals, the setting time can be more than 10 h.
iRoot SP (Sealer)	Innovative BioCeramix Inc., Vancouver, Canada	iRoot SP:zirconium oxide, calcium silicates, calcium phosphate, calcium hydroxide, filler and thickening agents	4 h
Tech Biosealer Endo	Isasan, Como, Italy	Mixture of white CEM, calcium sulphate, calcium chloride, bismuth oxide, montmorillonite	Tech Biosealer Endo has a final setting time of 77 min
EndoSeal MTA	Maruchi, Wonju, Korea	Calcium silicates, calcium aluminates, calcium aluminoferrite, calcium sulphates, radiopacifier and a thickening agent	12.31 min
MTA Fillapex	Angelus Industria de Produtos Odontologicos S/A, Londrina, Brazil	A MTA root canal sealer with nanoparticles of silica	The material’s setting time is 19.3 min. In dry conditions, the material fails to set
TheraCal LC (Light cured)	Bisco Inc., Schaumburg, IL, USA	CaO, Sr glass, fumed silica, barium sulphate, barium zirconate, Portland cement type III and resin containing Bis-GMA (bisphenol A-glycidyl methacrylate) and PEGDMA (polyethylene glycol-dimethacrylate)	The setting time has been reported to be 0.3 min because of the use of light cure technology
